# Linking songbird nest predation to seedling density: Sugar maple masting as a resource pulse in a forest food web

**DOI:** 10.1002/ece3.3581

**Published:** 2017-11-07

**Authors:** Marie‐Line Fiola, Alizée Vernouillet, Marc‐André Villard

**Affiliations:** ^1^ Département de biologie Université de Moncton Moncton NB Canada; ^2^ Department of Biological Sciences University of Manitoba Winnipeg MB Canada; ^3^ Département de biologie, chimie et géographie Université du Québec à Rimouski Rimouski QC Canada

**Keywords:** eastern deciduous forest, food web, indirect effects, migratory songbirds, nest predation, resource pulse

## Abstract

The ecological literature presents considerable evidence for top‐down forcing on the maintenance of species diversity. Yet, in temperate forests, bottom‐up forces often exert a strong influence on ecosystem functioning. Here, we report on the indirect influence of a pulsed resource, sugar maple (*Acer saccharum*) seed production, on nest survival in a migratory songbird. We hypothesized that seed production in year *t* would determine daily nest survival rate in year *t *+* *1 through its effects on seed‐eating rodents. We used the density of sugar maple seedlings (with cotyledons) in year *t *+* *1 as a proxy for seed production in year *t* and predicted that it would be inversely related to songbird nest survival the same year. We estimated the density of sugar maple seedlings, eastern chipmunk (*Tamias striatus*) activity, and daily nest survival rate in the ovenbird (*Seiurus aurocapilla*) over four successive years in a northern hardwood forest of New Brunswick, Canada. Seedling density varied by two orders of magnitude between years, whereas an index of chipmunk activity changed by an order of magnitude. Both variables were positively correlated and negatively correlated to daily nest survival rate. A logistic‐exposure model including only seedling density received the greatest level of support (lowest AIC_c_). Previous studies have reported the effect of sugar maple masting on seed‐eating rodent populations, but the strong link we report between seedling density and songbird nest survival is novel. A nocturnal seed‐eating nest predator, deer mouse (*Peromyscus maniculatus*), was not considered in our models, which may explain why chipmunk was not the best predictor of daily nest survival rate. The trophic linkages we observed are remarkably strong for a temperate forest ecosystem and might become more prevalent in northeastern North America, at least on calcium‐rich soils, with the loss of large‐diameter beech trees as a result of beech bark disease.

## 1 INTRODUCTION

Across a wide range of ecosystems, resource availability has been shown to vary through time and space, exhibiting rare, brief, and intense pulses (Yang, Bastow, Spence, & Wright, 2008). Resource pulses, that is, periods of abundant though intermittent availability of a resource in time and space, can also have a great influence on population dynamics and on the structure of species assemblages (Ostfeld & Keesing, 2000; Yang et al., 2008, 2010).

Pulsed resources have been reported in boreal forests dominated by spruces (*Picea* spp.) (Fletcher et al., 2010) and in temperate forests dominated by beeches (*Fagus* spp), yellow birch (*Betula alleghaniensis*), and especially oaks (Kelly, 1994; Koenig & Knops, 1998, 2000; McShea, 2000). In turn, masting (primary pulse) may initiate bottom‐up effects through primary consumers (e.g., rodents—secondary pulse), whose numerical response can have an indirect effect on the abundance of generalist predators (e.g., raptors or carnivores—tertiary pulse; Ostfeld & Keesing, 2000; Schmidt & Ostfeld, 2003; Clotfelter et al., 2007). For example, in deciduous forests of North America, acorn production has a direct effect on the density of mast‐consuming rodents the following spring (Jensen, Demers, McNulty, Jakubas, & Humphries, 2012; Schmidt, Rush, & Ostfeld, 2008; Schnurr, Ostfeld, & Canham, 2002). A positive relationship between seed production and rodent density has been documented in various forest stand types: oak‐hornbeam forest (Pucek, Jedrzejewski, Jedrzejewska, & Pucek, 1993); oak (Wolff, 1996); sugar maple (Falls, Falls, & Fryxell, 2007); and beech (King, 1983). Rodents can then significantly affect bird reproduction through predation on eggs, nestlings, or fledglings (Robertson, Hay, Saul, & McCormack, 1994; Schmidt & Ostfeld, 2003).

Tree masting appears to be largely controlled by weather conditions (Moreira, Abdala‐Roberts, Linhart, & Mooney, 2015; Pucek et al., 1993; Schauber et al., 2002; Smaill, Clinton, Allen, & Davis, 2011) and their effects on reproductive bud initiation, seed maturation (Houle, 1999), and pollen availability (Pensendorfer, Koenig, Pearse, Knops, & Funk, 2016). In several tree species, masting can be synchronized over hundreds of square kilometers (Koenig & Knops, 1998, 2000, 2013) and, in turn, spatially synchronized prey–predator–alternative prey relationships can be detected at scales up to ~1,000 km (Haynes et al., 2009).

In the eastern United States, the effects of acorn masting cycles on seed‐eating rodent populations and bird nest predation rates have been documented by McShea (2000), Clotfelter et al. (2007), Schmidt et al. (2008), and many others. In Canada, Falls et al. (2007) linked fluctuations in deer mouse abundance to sugar maple (*Acer saccharum*) masting, but they did not investigate the consequences of these fluctuations on alternative prey of mice, such as songbird nest contents (King & DeGraaf, 2006; Schmidt et al., 2001). Here, we report trophic relationships between sugar maple seed production, a seed‐eating rodent, and daily nest survival rate in a songbird. We focused on sugar maple masting as the trigger for small rodent population fluctuations because the other masting species present in our system (yellow birch and American beech, *Fagus grandifolia*) are unlikely to induce changes in rodent populations in our study area, whereas white ash (*Fraxinus americanus*) is nearly absent. Because of their very small size (0.12–0.24 g, DeHayes, Waite, & Hannah, 1980), yellow birch seeds appear unlikely to be stored by chipmunks for overwinter consumption, whereas seed production in the American beech is severely limited by beech bark disease (Nyland, Bashant, Bohn, & Verostek, 2006; Wagner et al., 2010), which is prevalent in our study area.

The eastern chipmunk (Tamias striatus), a seed‐eating nest predator, reaches very high abundances in years following mast seed production of certain tree species. We hypothesized that chipmunks will have a negative effect on the productivity of ground‐ and shrub‐nesting songbirds in years following sugar maple masting. One of those songbird species, the ovenbird (*Seiurus aurocapilla*), has been shown to be susceptible to nest predation by chipmunks (Reitsma, Holmes, & Sherry, 1990; King & DeGraaf, 2006; Appendix S1, Fig. S1). Observational (Morton, 2005) and experimental evidence (Emmering & Schmidt, 2011) also suggest that ovenbird females avoid nesting in areas of high eastern chipmunk activity. Here, we hypothesized that sugar maple seedling density (year *t *+* *1), a proxy for production of samaras in year *t*, will be positively correlated with ovenbird nest survival in year *t *+* *1 through the indirect effect of eastern chipmunk nest predation.

## 2 METHODS

### 2.1 Study area

**Figure 1:**
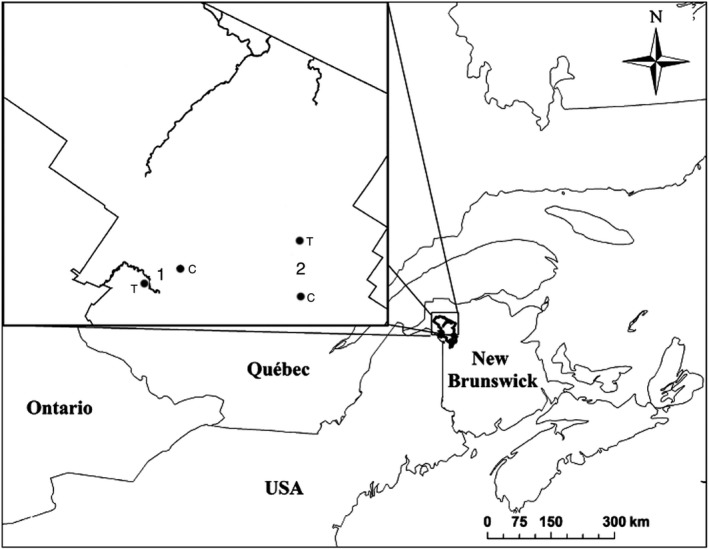
Location of study plots in the Black Brook District, in hardwood forest dominated by sugar maple (*Acer saccharum*), New Brunswick, Canada. Numbers indicate plot‐pairs, T indicates plots treated through selection harvesting (30%–40% basal area removal), and C indicates control plots

This study was conducted in the Black Brook District, a private managed forest covering approximately 2,000 km^2^ in northwestern New Brunswick, Canada (47°23′N, 67°40′W) (Figure 1). Study plots were selected as part of a before–after, control–impact paired (BACIP) design established in 2006 to quantify songbird demographic response to selection harvesting (Haché & Villard, 2010). Plots within pairs were located 3–6 km apart, and the mean distance between the five plot‐pairs was 23.8 km (±9.1 *SD*). For the purpose of this study, we focused on a subset of two of the five pairs of study plots (25 ha each) monitored by Haché and Villard (2010) over the 2012–2015 period, except when examining longer‐term trends in chipmunk abundance. During the winter of 2006–2007, one plot of each pair was treated through selection harvesting (30%–40% basal area removal). The four study plots (1T, 1C, 2T, and 2C; Figure 1) were located in well‐drained sites on glacial till deposits (Rampton, 1984), and they were dominated by shade‐tolerant deciduous tree species. In terms of relative basal area, the proportions of sugar maple, yellow birch, American beech, and other tree species (dbh > 10 cm) did not differ among the four plots (*G* = 10.5; *p *=* *.57). These plots were dominated by trees approximatively 120–150 years old and sugar maple was dominant, representing 66% of the total basal area, whereas yellow birch and American beech represented 19.7% and 7.8%, respectively (Table S1). Plot‐pair 1 was located on calcium‐rich soil and was characterized by a richer ground vegetation, including spring ephemerals (*Erythronium americanum, Claytonia caroliniana, and Dicentra cucullaria*; MAV, personal observation, see Hinds, 2000), that were absent from the other plot‐pair. Plant material on the ground including roots and leaves is an important part of chipmunk's diet (Wrazen & Svendsen, 1978).

### 2.2 Sampling of sugar maple seedlings

We inferred the previous year's sugar maple seed rain from the density of seedlings. This inference is based on several facts and some assumptions. First, under natural conditions, the viability of sugar maple seeds in the leaf litter does not exceed 1 year (Clayton, 1976). Seedling survival could also be influenced by factors such as herbivory or fungal infection (Cleavitt, Battles, Fahey, & Blum, 2014). However, we did not find information on seedling survival within the first days following germination in the literature and assumed that herbivory and fungal infection rate on newly emerged seedlings were relatively constant from year to year. We also assumed that germination rate was constant. According to Janerette (1979), germination rates of sugar maple seeds are high during cool, humid springs and those conditions are typical of springs in our study area (mean temperature = +10.9°C, *SD* = ±0.6; mean precipitation =110.7 mm, *SD* = ±22.0; data for May 2010–2014; Environment Canada, 2017). Finally, we assumed that chipmunk abundance would not have a significant effect on seedling density owing to a satiation effect (Fletcher et al., 2010; Linhart, Moreira, Snyder, & Mooney, 2014; Schnurr et al., 2002).

We estimated the density of newly emerged seedlings (with cotyledons) in each 25‐ha plot by counting them in 50 quadrats (50 cm × 50 cm each) placed at randomly selected locations that were at least 25 m apart. Seedling counts were conducted between 22 and 29 May each year, except in 2012 (30‐May – 9‐June).

### 2.3 Nest predator surveys

Considering the size of study plots, it would have been impractical to conduct live trapping. Instead, we recorded all chipmunk detections, whether visual or aural, using a protocol inspired from avian spot‐mapping. We also recorded detections of red squirrel (*Tamiasciurus hudsonicus*) and blue jay (*Cyanocitta cristata*) because all three species are known predators on bird nests, including those of ovenbirds, a common ground‐nesting songbird (King & DeGraaf, 2000; Porneluzi, Van Horn, & Donovan, 2011). Because chipmunks were by far the most common of the three, with number of detections per survey an order of magnitude higher, we only used chipmunk detections for analytical purposes.

Each plot was surveyed between 1000 and 1300, a period encompassing the peak of chipmunk activity (Snyder, 1982). We conducted six surveys per plot each year, between 23 May and 21 June, except in 2013, when only five visits were completed due to unusually cold weather at the end of May. Because chipmunks are less active in cold weather (Dunford, 1972), we assumed that the later start did not substantially change the mean chipmunk activity index we recorded that year. Surveys were only conducted under favorable conditions (i.e., no reduction in detectability due to precipitation or wind). Observers walked slowly, at approximately 5 km/h, along transects located 50 m apart and recorded all visual or aural detections within a 25‐m band on either side of the transect on a detailed map of the grid. Transect length varied depending on plot shape, but survey time remained constant in each plot (approximately 4 hours). The entire area of each plot (25 ha) was gridded. Precautions were taken to prevent double‐counting of individuals. For example, two detections within 5 m of each other recorded along adjacent transects were considered as a single individual. The starting point alternated between surveys to encompass all chipmunk activity during the peak activity period. Most detections were visual, sometimes following an aural detection. We restricted detection distance to 25 m to maximize detectability. When analyzing each year separately, we found no observer effect on the number of detections (Appendix S2, Table S2).

### 2.4 Ovenbird nesting success

We mapped ovenbird territories using a standard spot‐mapping method (Bibby, Burgess, Hill, & Mustoe, 2000). Observers walked along transects located 100 m apart, stopping every 25 m, and recorded all detections on a map within a 50 m band on either side. Each year (25‐May – 30‐June), we conducted eight spot‐mapping visits per plot between 0600 and 0930. Surveys were only conducted under favorable weather (i.e., no reduction in detectability due to precipitations or strong winds). On the basis of mapped territories, we searched intensively for nests and monitored all active nests every 3 days until depredated, abandoned, or all young had fledged. When monitoring nests, we followed Martin and Geupel's (1993) protocol and used extra care not to flush the female unnecessarily. First, nests were found between 22 May and 15 June, and nest searching was pursued until the end of July each year. Nests were found either by following females, by flushing them from their nest, or through visual scanning. A nest was considered successful if it fledged at least one young or if nestlings were at least 8 days old at the last visit and the nest was later found empty and undisturbed. Nests with obvious signs of predation (eggshell fragments, damaged nest structure) or whose nestlings had disappeared before they were 8‐days old were considered depredated. Nests containing eggs that were left unattended over at least 9 days were considered abandoned. We have used this method to estimate ovenbird nesting success in the five pairs of plots since 2006, including the two pairs that were used to estimate maple seedling density and chipmunk density. During songbird spot‐mapping, we also recorded detections of potential nest predators in all plots. Although these surveys were less intensive than chipmunk surveys (50 m survey bands compared to 25 m), this allowed us to detect major peaks in nest predator activity between 2006 and 2015.

### 2.5 Statistical analyses

All statistical analyses were performed using R version 3.2.2 (R Core Team, 2015). We used logistic‐exposure models (Shaffer, 2004) and binomial distribution to evaluate competing models of daily survival rate (DSR) of nests. These models allow varying length of observation interval (in days) for nests. Interval data were compiled following Shaffer (2004). For this analysis, we excluded nests that were abandoned or whose fate was unknown. Abandonment usually occurred prior to the laying of the first egg and, therefore, there were no days of exposure for those nests. We estimated nest survival using a 27‐day nest period (i.e., from first egg laid to fledging; Porneluzi et al., 2011). Models were constructed to determine whether DSR of nests was influenced by continuous (chipmunk activity index and seedling density) and categorical (year, treatment, plot, and plot‐pair) explanatory variables (Table 1). We included interactions (chipmunks × year and chipmunks × plot) to determine whether the effect of chipmunk activity varied between years or among plots. Considering that multicollinearity was low between chipmunks and seedlings (VIF=1.02), we also considered an additive model with those two predictor variables. We constructed 21 a priori models corresponding to hypothesized relationships among variables, as well as a null model (intercept only). For model selection, we used an information‐theoretic approach (Burnham & Anderson, 2002) based on second‐order Akaike's Information Criterion (AIC_c_), corrected for small sample size using AICcmodavg package (Mazerolle, 2015) in R. We applied natural log transformations to seedling density [ln(seedlings + 1)] and chipmunk activity index [ln(chipmunks + 2)] to normalize their distribution. We used model averaging in order to incorporate model uncertainty into the estimates of parameters (Doherty, White, & Burnham, 2012), and the natural average method (Burnham & Anderson, 2002) to further examine the variables of interest (here, chipmunks and seedlings) (Grueber, Nakagawa, Laws, & Jamieson, 2011). In the natural average method, the estimate for each predictor is averaged among the models including the variable of interest and the Akaike weights are recalculated to determine a weighted average of the parameter estimate. Evidence ratios were calculated by dividing the weight of evidence for all models including the effect of interest by the weight of evidence for all equivalent models without the effect of interest. In order to be fully AIC compatible, we computed unconditional 85% confidence intervals for the parameters of interest, as AIC‐based model selection will support variables whose 85% confidence intervals exclude zero (Arnold, 2010).

**Table 1:** Variables used in logistic‐exposure models to assess their influence on daily survival rate of ovenbird nests (*n* = 53) in New Brunswick, Canada

Parameters	Code	Predicted effect on DSR
Chipmunk activity index	Chipmunks	Negative
Seedling density	Seedlings	Negative
Year	Year	–
Selection harvesting treatment	Treatment	Unknown
Plot	Plot	–
Plot‐pair^a^	Plot‐pair	–

a Plots were spatially paired (one treated plot and one control plot per pair), see Methods.

## 3 RESULTS

### 3.1 Sugar maple seedlings and eastern chipmunks

**Figure 2:**
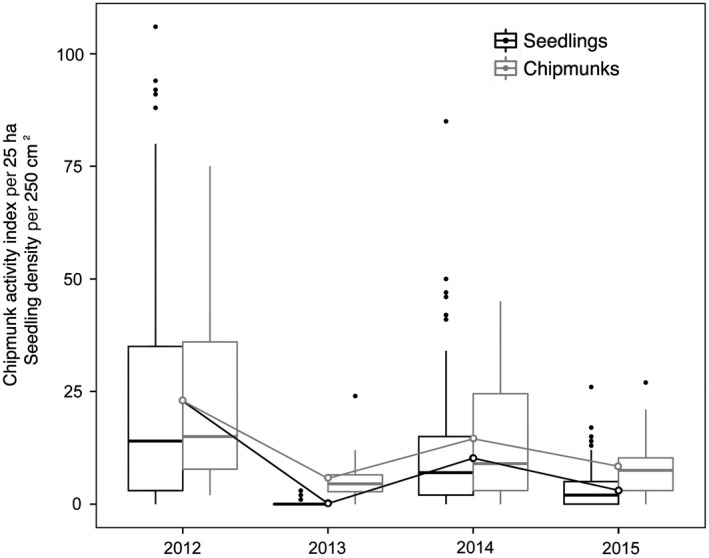
Year‐to‐year variations in sugar maple seedling density and eastern chipmunk activity index. Open symbols represent means across all plots

**Figure 3:**
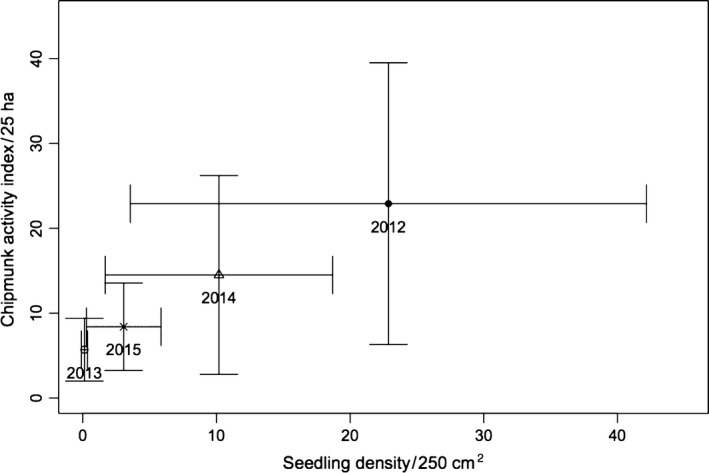
Relationship between eastern chipmunk activity index (mean detections across plots) and density of sugar maple seedlings per year

Seedling density and chipmunk detections varied considerably among years (Figure 2). The relationship between mean seedling density and mean chipmunk activity was positive and linear, whether data were examined on a per plot basis (Appendix S1, Fig. S2) or pooled by year (Figure 3). However, the small sample size per plot prevented the calculation of correlation coefficients. Chipmunk activity was an order of magnitude higher in a pair of plots (1T–1C) located on calcium‐rich soils (see Methods).

Lower chipmunk activity in plot 2C might reflect the presence of a Northern Goshawk (*Accipiter gentilis*) pair that nested within or near the plot each year (see Smithers, Boal, & Andersen, 2005).

### 3.2 Nest survival rate

**Figure 4:**
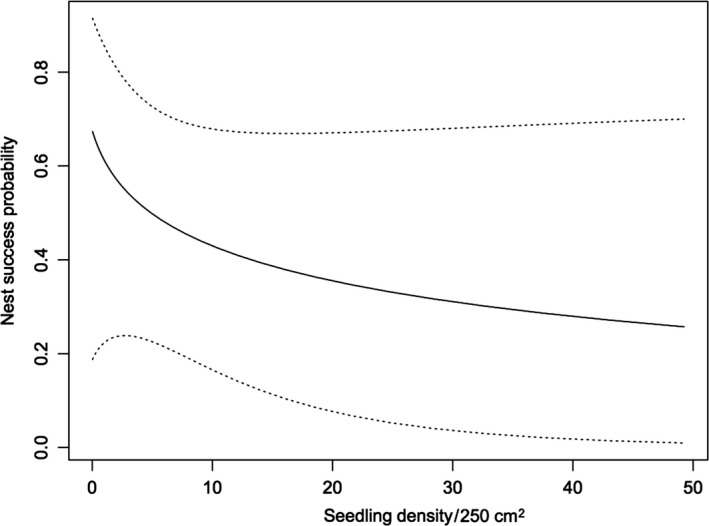
Predicted nest success probability in the ovenbird in relation to seedling density based on natural multimodel averaging of all candidate logistic‐exposure models. Dashed lines represent 95% confidence intervals

In the four study plots, we found a total of 69 ovenbird nests over the 4 years. Of these, 28 were successful, 25 were depredated, and 16 were abandoned. Among the a priori models tested to explain DSR of nests, the one that only contained the variable seedlings was ranked highest and had an AIC_c_ weight of 0.23 (Table 2). We found substantial evidence for a seedling effect (evidence ratio = 2.1), but less evidence for a chipmunk effect (evidence ratio = 0.6). Natural model‐averaged parameters indicate that seedlings had an important negative, indirect effect on DSR, whereas chipmunks had no effect (Table 3). Because the seedlings variable was only present in models with relatively high weights, we can safely assume that parameter estimates were not biased by natural model averaging. Model‐averaged predictions based on the entire candidate model set are shown in Figure 4.

**Table 2:** Performance of 21 logistic‐exposure models predicting daily survival rate of ovenbird nests (*n* = 60) in New Brunswick, Canada (2012–2015), with number of parameters (*K*), difference in Akaike Information Criterion values (corrected for small sample size) between each model and the top model (ΔAIC_c_), and Akaike weight (w_i_)

DSR Models	*K*	AIC_c_	ΔAIC_c_	w_i_
Seedlings	2	86.47	0.00	0.23
Seedlings + Plot‐pair^a^	3	87.36	0.88	0.15
Seedlings + Chipmunks	3	87.92	1.45	0.11
Chipmunks	2	88.33	1.86	0.09
Seedlings + Treatment^b^	3	88.72	2.25	0.07
Null	1	89.40	2.93	0.05
Chipmunks + Treatment	3	89.59	3.11	0.05
Chipmunks × Plot	8	89.69	3.22	0.05
Seedlings + Plot	5	89.82	3.34	0.04
Plot‐pair	2	90.22	3.74	0.04
Chipmunks + Plot‐pair	3	90.25	3.78	0.03
Treatment	2	90.92	4.44	0.02
Plot	4	91.17	4.70	0.02
Treatment + Plot‐pair	3	91.72	5.24	0.02
Chipmunks + Plot	5	91.78	5.30	0.02
Chipmunks + Year	5	93.06	6.58	0.01
Year + Pair‐plot	5	93.39	6.92	0.01
Year + Plot	7	93.47	6.99	0.01
Year	4	Did not converge		
Seedlings + Year	5	Did not converge		
Chipmunks × Year	8	Did not converge		

a Plots were spatially paired (one treated plot and one control plot per pair), see Methods.

b Refers to the effect of selection harvesting in 2006 (30%–40% basal area removal).

**Table 3:** Model‐averaged parameter estimates for explanatory variables included in competing logistic‐exposure models (ΔAIC_c_ ≤ 2.0) predicting daily nest survival rate in the ovenbird, New Brunswick, Canada, 2012–2015

Explanatory variables	Model‐averaged estimate	Unconditional SE	85% Unconditional confidence interval	
			Lower	Upper
Seedlings	−0.5	0.26	−0.88	−0.12
Chipmunks	−0.5	0.42	−1.09	0.13

**Figure 5:**
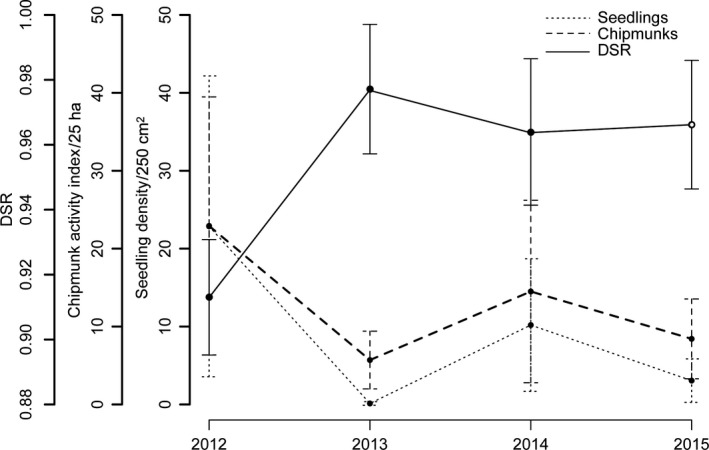
Variations in daily survival rate (DSR) of ovenbird nests, seedling density, and an index of eastern chipmunk activity over the duration of the study (2012–2015). Chipmunk activity index (chipmunks) and seedling density (seedlings) were calculated in plot‐pairs 1 and 2. DSR was calculated for the five plot‐pairs (see Figure 1) in 2012, 2013, and 2014 (filled dots) and plot‐pairs 1 and 2 in 2015 (open dot)

Sugar maple masting is known to occur over very large spatial scales, for example, across southern Québec (D. Lévesque, personal communication). Assuming constant DSR for ovenbird nests across the broader study area (i.e., all five pairs of plots), both seedling density and chipmunk activity peaked in 2012, whereas DSR was the lowest (0.913, 95% CI 0.895–0.931), corresponding to a mere 8.5% nest survival over the duration of the nesting period (27 days). Percent nest abandonment also peaked in 2012 (57%), compared to 18% in 2013, 10% in 2014, and 30% in 2015. In 2013, both seedling density and chipmunk activity were lowest, whereas DSR peaked (0.977, 95% CI 0.957–0.997), corresponding to a 53.6% nesting success. In 2014 and 2015, values for all three variables were intermediate.

## 4 DISCUSSION

Seedling density was a good predictor of ovenbird nest survival rate the same year, as hypothesized. Chipmunk activity, however, received limited support. The relationship between seedling density and chipmunk activity in a given year was positive and linear, although slopes varied strongly among plots. In contrast, Schnurr et al. (2002) did not detect a relationship between chipmunk abundance and the seed rain of any tree species in their Connecticut study area, probably as a result of a greater diversity of tree species.

Seedling density had an important indirect negative effect on daily survival rate of nests. In a system driven by acorn masting, Schmidt et al. (2008) reached a similar conclusion: wood thrush (*Hylocichla mustelina*) nest survival rate did not vary as a function of rodent abundance the same year, but was rather related to acorn production the previous year. Leimgruber, McShea, and Rappole (1994) and McShea (2000) also found that masting crops of the previous fall, not a given year's density of seed‐eating rodents, were a significant predictor of nest predation rate. However, they used artificial nests and the resulting nest predation rates may not closely reflect those of nearby, natural nests (Burke et al., 2004; Moore & Robinson, 2004). Schmidt and Ostfeld (2008) found a positive correlation between rodent abundance and nest predation rate in the veery (*Catharus fuscescens*), a ground‐nesting species, but not in the shrub/subcanopy‐nesting wood thrush. Here, we suspect that the influence of another seed‐eating nest predator could explain why chipmunk activity was not as good a predictor as seedlings. Deer mouse also responds numerically to sugar maple masting (Falls et al., 2007) and can depredate songbird nests in a density‐dependent fashion (Bradley & Marzluff, 2003). Hence, mice might be responsible for some of the predation events in our system. Female ovenbirds have been observed defending their nest against mice, but the latter eventually ate the eggs or nestlings (King & DeGraaf, 2006). Unfortunately, we did not monitor deer mouse abundance, but as mentioned above, years of high chipmunk activity probably coincided with peaks in deer mouse abundance, as observed by Fryxell, Falls, Falls, and Brooks (1998) elsewhere in the northern hardwoods forest.

The wide variations we observed in sugar maple seedling density are consistent with studies that monitored seed production in this species over several years (Cleavitt & Fahey, 2017; Falls et al., 2007; Houle, 1999; Jensen et al., 2012; Schnurr et al., 2002). Seedling density appeared to be a good indicator of the previous year's seedfall, which was high in the adjacent region of Bas‐Saint‐Laurent, Québec in 2006 and 2011, followed by very low and intermediate crops in 2012 and 2013, respectively (D. Lévesque, personal communication). From 2006 to 2015, our spot‐mapping data set (see Methods) indicated that chipmunk activity varied considerably and the peaks we detected in 2007 and 2012 (Fig. S3) appeared to coincide with sugar maple seedfall the previous year. The same trend was also observed in sugar maple seedfall in northern hardwood forests of New Hampshire, with peaks in seedfall in 2006 and 2011 (Cleavitt & Fahey, 2017). This strongly suggests that sugar maple masting is synchronized over very large portions of its range. In years following low seed crops, adult chipmunks are less active and mainly stay underground (Bergeron, Réale, Humphries, & Garant, 2011), which would explain the lower chipmunk activity detected in 2013 (Appendix S1, Fig. S3). This pattern has been shown elsewhere. What is truly novel here is the link we found between the density of a new seedling cohort and nest survival rate in a songbird, irrespective of the nest predator species involved.

In addition to time‐lagged, bottom‐up effects associated with mast‐seeding trees, songbird population growth rate can be lower in years of low rodent abundance (Schmidt & Ostfeld, 2008). During rodent population crashes, top predators, driven by a functional and numerical response to primary prey abundance (e.g., rodents; Ostfeld & Keesing, 2000; Jensen et al., 2012), can increase predation on songbird nests and fledglings as a result of a diet shift (McShea, 2000). Hence, pulsed resources may initiate bottom‐up effects that are followed by top‐down trophic cascades (Pace, Cole, Carpenter, & Kitchell, 1999). We did not monitor the abundance of, nor did we have access to data on top predators in our system, such as barred owl (*Strix varia*), American marten (*Martes americana*), or fisher (*M. pennanti*). Our time‐series was also too short to investigate such complex effects.

Compared to arctic ecosystems that are often considered to have simple food webs (Gauthier et al., 2011; Legagneux et al., 2012), temperate deciduous forests have typically more complex food webs, with up to two or three species of trees exhibiting masting cycles (Graber & Leak, 1992), and many seed‐eating species responding to those pulses (Jensen et al., 2012; Schmidt & Ostfeld, 2008). This is the first time, to our knowledge, that sugar maple appears to act as a resource pulse modulating populations of seed‐eating rodents and, in turn, songbird nest survival.

Population growth rate might not be directly linked to nest survival. Streby and Andersen (2011) argued that population growth rate should not be estimated on the basis of nest data, but rather on direct estimates of juvenile survival. However, unlike other regions where avian predators play an important role (Streby & Andersen, 2011), ovenbird postfledging survival rate appears to be strongly tied to sciurid abundance (i.e., eastern chipmunk and red squirrel) in our study area (Haché, Bayne, & Villard, 2014). Hence, sugar maple masting would be expected to affect juvenile survival rate, and thus population growth rate, through its influence on sciurid abundance (see also Schmidt et al., 2008).

The generality of our findings will depend on the effects of beech bark disease on mast‐producing tree species composition in hardwood forests of northeastern North America. With the expansion of the disease, the role of sugar maple masting in trophic dynamics may become more prevalent throughout the northern portions of the eastern deciduous forest, where oaks are generally uncommon (Canada's National Forest Inventory, 2013). Beechnuts provide a major food source for chipmunks and many other seed‐eating nest predators (Bergeron et al., 2011; Rosemier & Storer, 2010), and beech bark disease can potentially reduce beech mast volumes (Nyland et al., 2006; Wagner et al., 2010). In fact, it is difficult to compare nut production before and after beech bark disease because no quantitative data are available (McNulty & Masters, 2005). Surprisingly, some studies have reported that beech nut production was highest in plots with the greatest intensity of beech bark disease (McNulty & Masters, 2005; Garneau et al., 2012). The disease appears to stress larger trees, which respond by increasing their reproductive investment (Hagen, Folstad, & Jakobsen, 2003). However, Lovett, Arthur, Weathers, and Griffin (2013) have shown that premature mortality of beech trees can provoke a significant shift in tree species composition, favoring sugar maple on calcium‐rich soils. Thus, the link between sugar maple seed production, seed‐eating rodents, and songbirds could potentially become as important as the acorn‐driven resource pulses in oak‐dominated deciduous forests further south.

## CONFLICT OF INTEREST

None declared.

## AUTHOR CONTRIBUTIONS

MLF, AV, and MAV conceived the ideas and designed methodology; MLF, AV, and MAV collected the data; MLF analyzed the data; MLF, AV, and MAV interpreted the results; MLF and MAV led the writing of the manuscript. All authors contributed critically to the drafts and gave approval for submission.
