# Supplementary material for: Linking songbird nest predation to seedling density: Sugar maple masting as a resource pulse in a forest food web
Source: Ecol Evol. 2017 Nov 7;7(24):10733–42. doi: 10.1002/ece3.3581 (PMC5743542; doi:10.1002/ece3.3581)
Supplement: Supplementary file 1 [file ECE3-7-10733-s001.docx]

**Appendices**

Appendix S1.

Fig. S1. Activity around an ovenbird nest monitored using an infrared-triggered camera. The nest entrance is facing away from the camera, near the middle of the image. (A) Female bringing food to her nestlings; (B) eastern chipmunk at the nest entrance

(A)


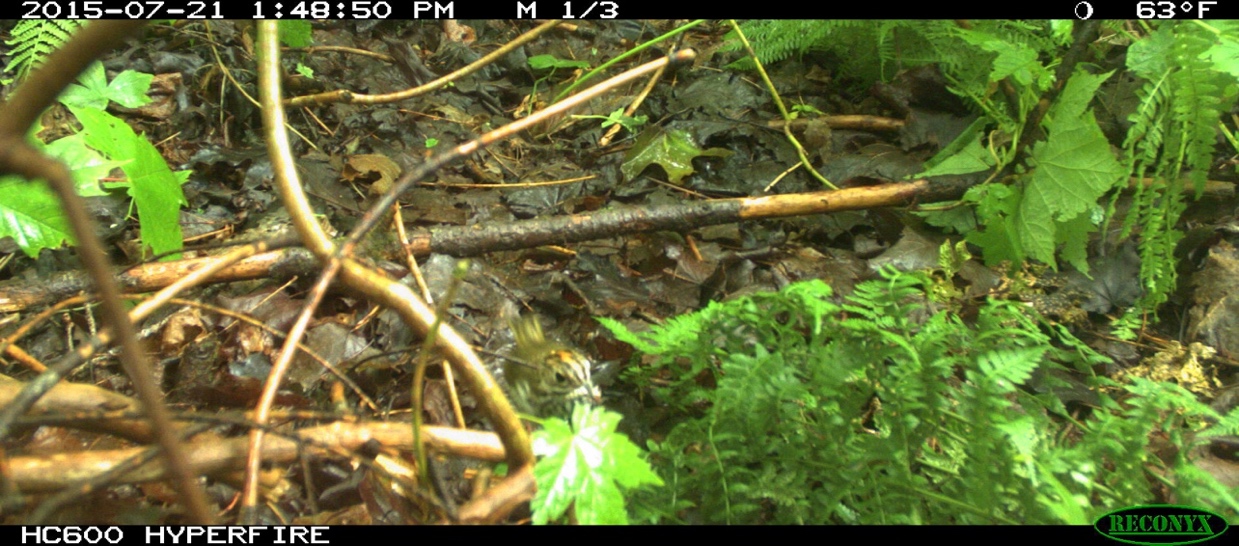


(B)


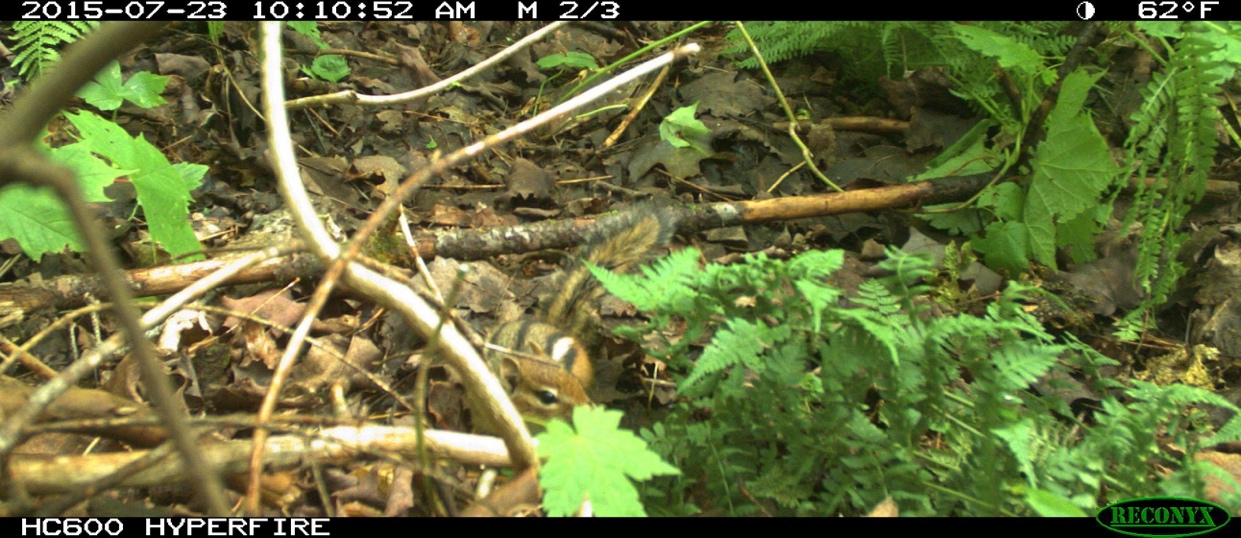


Fig. S2. Relationship between eastern chipmunk activity index (mean number of detections per plot) and density of sugar maple seedlings in each study plot across years. See Figure 1 for meaning of plot codes.


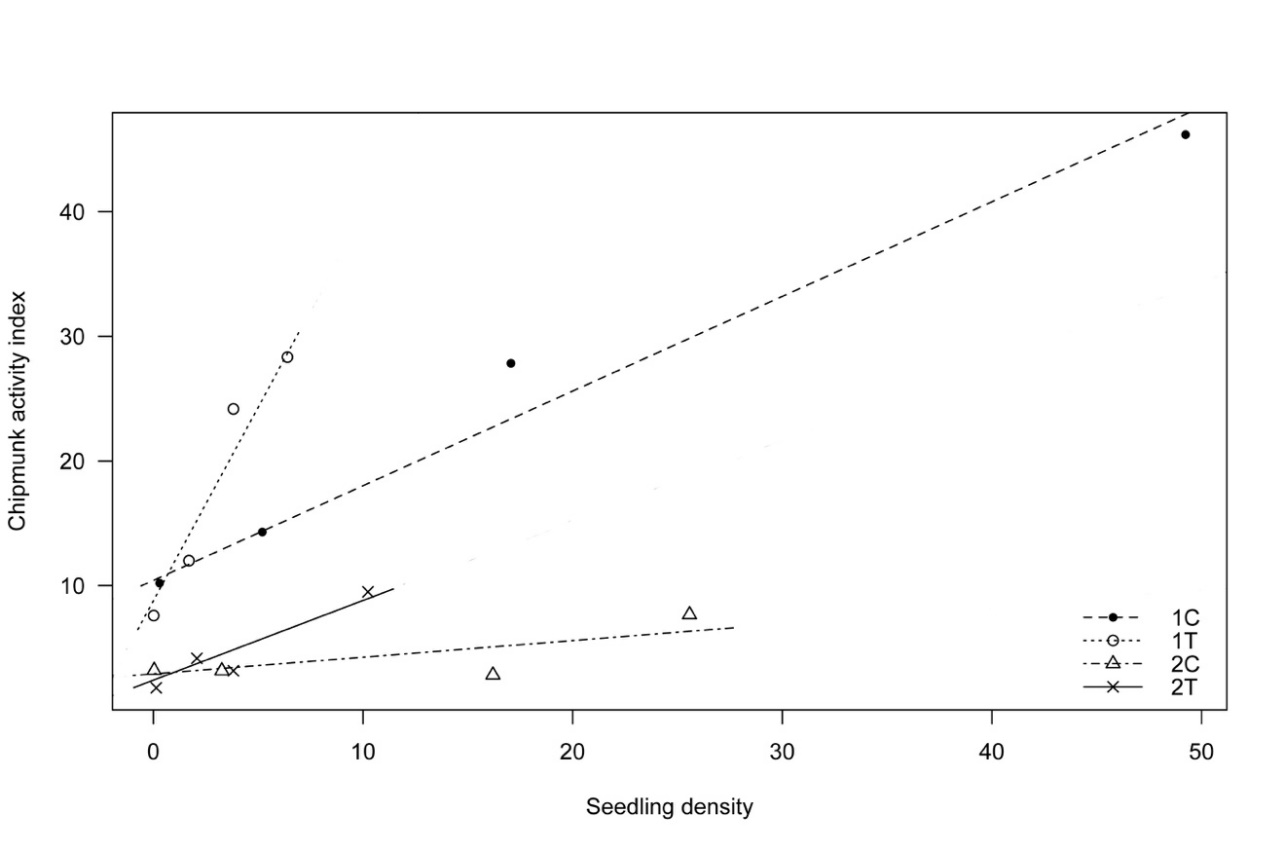


Fig. S3. Index of eastern chipmunk activity (±SE) based on the mean number of detections per avian spot mapping visit for the 5 plot pairs from 2006 to 2014 (filled dots) and plot pairs 1 and 2 in 2015 (open dot).


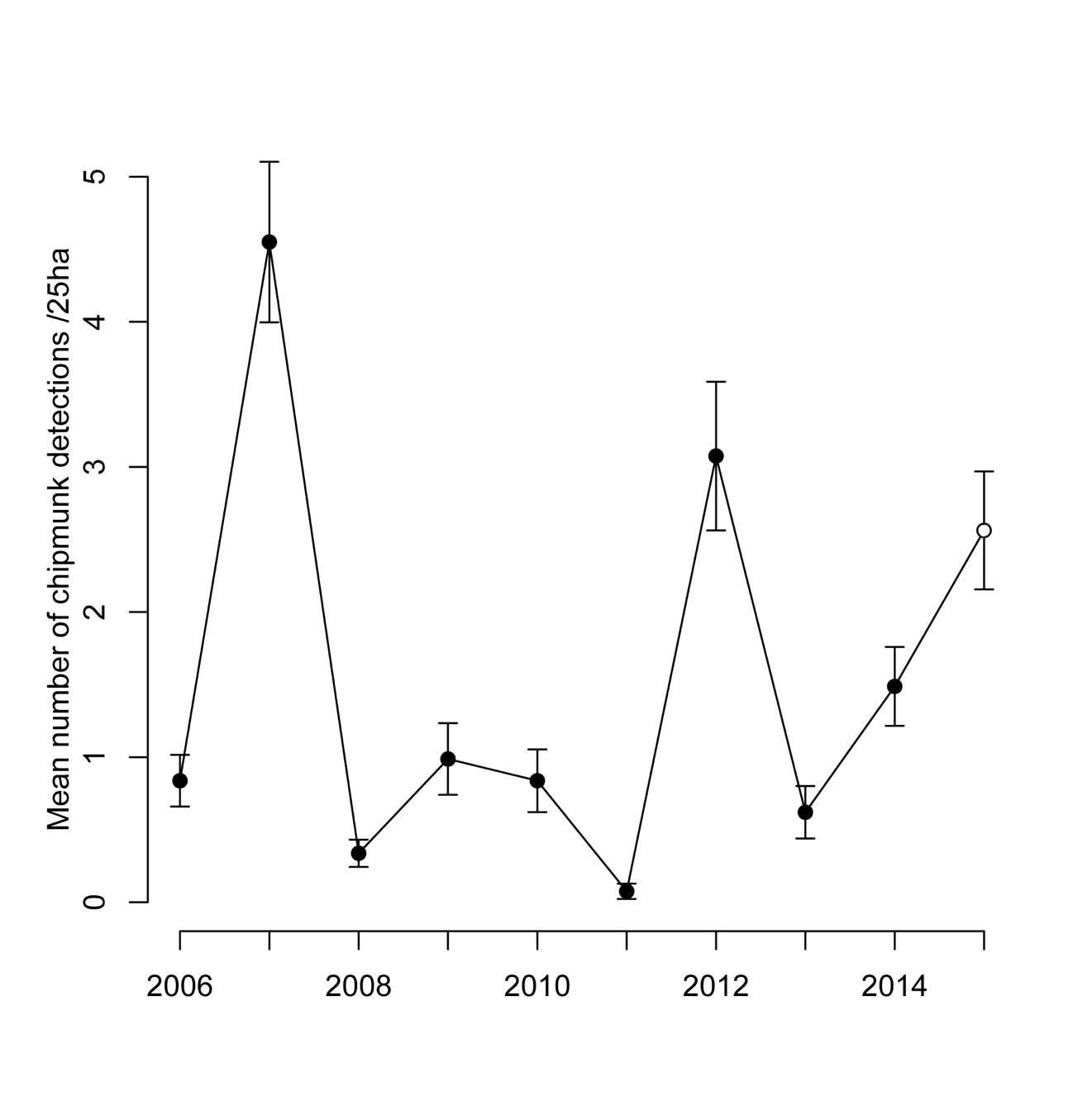


Appendix 2.

Table S1. Percent basal area of the three dominant tree species (dbh ≥10 cm) in each study plot (25 ha each), in northwestern New Brunswick, Canada (2014). We compared relative basal areas using a G-test of independence.

|  | Study Plot | | | | |
| --- | --- | --- | --- | --- | --- |
| Tree species | 1T | 1C | 2T | 2C | All Plots |
| Sugar maple | 66.46 | 85.56 | 52.29 | 63.09 | 66.45 |
| Yellow birch | 17.09 | 6.70 | 29.96 | 21.32 | 19.70 |
| American beech | 7.28 | 7.46 | 10.18 | 6.21 | 7.75 |
| Others | 9.17 | 0.28 | 7.57 | 9.38 | 6.10 |

Table S2. Comparison of number of the chipmunk detections recorded per observer, analysed for each year separately. Results suggest no significant observer effect.

| Year | Kruskal-Wallis chi-squared | df | p-value |
| --- | --- | --- | --- |
| 2012 | 3.3819 | 2 | 0.184 |
| 2013 | 1.7398 | 2 | 0.419 |
| 2014 | 0.0053 | 2 | 0.974 |
| 2015 | 10.003 | 5 | 0.075 |
